# Anthropometry, body shape in early-life and risk of premenopausal breast cancer among Latin American women: results from the PRECAMA study

**DOI:** 10.1038/s41598-020-59056-6

**Published:** 2020-02-10

**Authors:** Mathilde His, Carine Biessy, Gabriela Torres-Mejía, Angélica Ángeles-Llerenas, Isabel Alvarado-Cabrero, Gloria Inés Sánchez, Mauricio Borrero, Carolina Porras, Ana Cecilia Rodriguez, Maria Luisa Garmendia, Magali Olivier, Peggy L. Porter, MingGang Lin, Marc J. Gunter, Isabelle Romieu, Sabina Rinaldi, PRECAMA team, Jenny Tejeda, Edgar Navarro, Roberto Jaramillo, Yorlany Rodas Cortes, Alberto Angel, Carlos Andres Ossa, William H. Arias, Gabriel Bedoya, Alicia Maria Cock-Rada, Carolina Echeverri, Fernando Herazo, Israel Díaz-Yunez, Angel Hernández, Bernal Cortes, Paula Gonzalez, Rebecca Ocampo, Diego Guillen, Leonor Moyano, Jose Luis Soto, Elizabeth Donato, Jamie Guenthoer, Thomas Donn, Kelly Wirtala, Hailey Loucks

**Affiliations:** 1 Section of Nutrition and Metabolism, International Agency for Research on Cancer, Lyon, France; 2 Centre for Population Health Research, National Institute of Public Health, Cuernavaca, Mexico; 3 Servicio de Patología del Hospital de Oncología, Centro Médico Nacional Siglo XXI, Instituto Mexicano del Seguro Social, Mexico city, Mexico; 4 Group Infection and Cancer, School of Medicine, University of Antioquia, Medellín, Colombia; 5 Department of Gynecology and Obstetrics, School of Medicine, University or Antioquia, Medellín, Colombia; 6 Cinica Vida Fundacion, Medellín, Colombia; 7 Agencia Costarricense de Investigaciones Biomédicas (ACIB)-Fundación INCIENSA, San José, Costa Rica; 8 Instituto de Nutrición y de Tecnología de los Alimentos, Universidad de Chile, Santiago, Chile; 9 Molecular Mechanisms and Biomarkers Group, Section of Mechanisms of Carcinogenesis, International Agency for Research on Cancer, Lyon, France; 10 Division of Human Biology, Fred Hutchinson Cancer Research Center, Seattle, United States of America; 11 Hubert Department of Global Health, Emory University, Atlanta, Georgia United States of America; 12 Grupo Proyecto UNI-Barranquilla, Universidad del Norte, Barranquilla, Colombia; 13 Hemato Oncologos, Cali, Colombia; 14 Torre Médica Las Américas, Medellín, Colombia; 15 Torre Médica El Tesoro, Medellín, Colombia; 16 GENMOL Group, Natural and Basic Sciences Faculty, University of Antioquia, Medellín, Colombia; 17 Instituto de Cancerología Las Américas, Medellín, Colombia; 18 Imágenes Diagnósticas y Biotecnología Reproductiva, Cediul S.A., Barranquilla, Colombia; 19 Clínica Bonnadona Prevenir, Barranquilla, Colombia; 20 National Institute of Cancer, Santiago, Chile

**Keywords:** Breast cancer, Cancer epidemiology, Cancer prevention, Risk factors

## Abstract

Cumulating evidence in Caucasian women suggests a positive association between height and premenopausal breast cancer risk and a negative association with overall adiposity; however data from Latin America are scarce. We investigated the associations between excess adiposity, body shape evolution across life, and risk of premenopausal breast cancer among 406 cases (women aged 20–45) and 406 matched population-based controls from Chile, Colombia, Costa Rica, and Mexico. Negative associations between adult adiposity and breast cancer risk were observed in adjusted models (body mass index (BMI): Odds ratio (OR) per 1 kg/m^2^ = 0.93; 95% confidence interval = 0.89–0.96; waist circumference (WC): OR per 10 cm = 0.81 (0.69–0.96); hip circumference (HC): OR per 10 cm = 0.80 (0.67–0.95)). Height and leg length were not associated with risk. In normal weight women (18.5 ≤ BMI < 25), women with central obesity (WC > 88 cm) had an increased risk compared to women with normal WC (OR = 3.60(1.47–8.79)). Residuals of WC over BMI showed positive associations when adjusted for BMI (OR per 10 cm = 1.38 (0.98–1.94)). Body shape at younger ages and body shape evolution were not associated with risk. No heterogeneity was observed by receptor status. In this population of Latin American premenopausal women, different fat distributions in adulthood were differentially associated with risk of breast cancer.

## Introduction

Although breast cancer is the most commonly diagnosed cancer among women worldwide^1^, the age distribution of the cases varies between different regions of the world. While only 12.4% of the women diagnosed with breast cancer in 2018 were younger than 45 years old in high-income countries, this proportion reached 21% in Latin America^1^. It has been shown that only part of this difference can be attributed to the difference in the age structure of the population^2^, suggesting a possible role for specific etiologic factors. However, to date only a few studies have investigated risk factors for premenopausal breast cancer among women in Latin America^3^.

Studies from Caucasian populations indicate that the risk of breast cancer in premenopausal women increases with height^4,5^ and decreases with excess adiposity in adulthood^4^, as well as during childhood/puberty, although with less consistent results^6,7^. These associations are observed mostly for hormone receptor positive tumors^5,8^. However, only one study has so far been conducted in women in Latin America^9^, which suggested associations similar to those in Caucasian women but did not examine associations by subtypes. Given the rapidly growing burden of obesity in Latin America, where the mean body mass index (BMI) in women increased from 23 kg/m^2^ in 1980 to 27 kg/m^2^ in 2016^10^, assessing the association between obesity and breast cancer risk is crucial.

In this study, we investigated the associations between several anthropometric factors assessed during adulthood, and changes in body shape during the life course, with the risk of premenopausal breast cancer, overall and by subtypes. This study was designed within the framework of the PRECAMA project, an ongoing multicentric population-based case-control study initiated in four countries in Latin America, coordinated by the International Agency for Research on Cancer (IARC).

## Methods

### The PRECAMA study

PRECAMA is an ongoing multicentric population-based case-control study based in Chile, Colombia, Costa Rica, Mexico, and Brazil (pilot phase)^11–13^. At recruitment, information was collected on lifestyle, health and reproductive history. Diet was assessed using a standardized food-frequency questionnaire that included country-specific foods. Standardized and harmonized protocols were used to perform anthropometric measures and to collect biological samples (fasting blood, spot urine) at the time of the interview. Immunohistochemistry analyses on tumor tissue for estrogen receptor (ER), progesterone receptor (PR), human epidermal growth factor receptor 2 (HER2), epidermal growth factor receptor (EGFR) and cytokeratin 5/6 (CK5/6) was performed centrally at the Fred Hutchinson Cancer Research Center (FHCRC) in Seattle. All participants gave written informed consent before enrolment, and the study protocols were approved by the institutional review boards of Chile (Oncologic Institute Foundation Arturo Lopez Pérez, Instituto de Nutrición y de Tecnología de los Alimentos, National Cancer Institute), Colombia (Cancer Institute Las Americas and University of Antioquia), Costa Rica (Costa Rican Institute of Clinical Research (ICIC) and Center for Strategic Development and Information in Health and Social Security (CENDEISSS) of the Costa Rican Social Security Fund (CCSS)), Mexico (National Institute of Public Health and the Social Security Mexican Institute), and the International Agency for Research on Cancer (IARC). All methods were performed in accordance with the relevant guidelines and regulations of these approvals.

### Selection of cases and controls

Incident cases of primary invasive breast cancer were recruited from general or cancer-specific hospitals or private oncology institutes, encompassing populations with a wide range of socio-economic status^13^. The inclusion criteria were: age 20–45 years; being resident for ≥3 years in the same city district; having an incident primary invasive BC with positive biopsy and clinical staging and having menstruated at least once in the past 12 months. The exclusion criteria were having severe chronic disease, limited ability to communicate, being pregnant or nursing and having previous diagnosis and treatment of cancer (except non-melanoma skin cancer). Cases were recruited before any treatment and after signing an informed consent to participate.

Controls were selected from the general population residing in the same city district as the case for at least 3 years using a multilevel sampling frame, applying the same exclusion criteria as for the cases^13^. Controls were matched to cases on age (±3 years), city district of residence, and health insurance institution. The current analysis includes 406 cases and 406 matched controls.

### Pathology review and immunohistochemical analyses

Histology sections from paraffin-embedded tumor biopsies obtained before any treatment or obtained during surgery if no adjuvant therapy was administered, were reviewed for diagnosis, tumor grade, lymph vascular invasion and stromal and lymphocyte response in a centralized laboratory in Seattle, USA (the Porter Lab, FHCRC). Tumor samples with more than 1% immunostained tumor cell nuclei were considered ER-positive (ER+) and PR-positive (PR+). For HER2, samples were considered positive if there was strong membrane immunostaining (3+) and negative otherwise. Triple-negative (TN) tumors were defined as ER−, PR-, and HER2-. Among the TN tumors, basal-like cancers were defined as ER−, PR−, HER2−, and EGFR-positive (EGFR+) and/or CK5/6-positive (CK5/6+).

### Body size assessment

At recruitment, trained clinicians conducted anthropometric measurements according to standardized protocols and following Lohman’s recommendations^14^. Height, sitting height, weight, and waist (WC) and hip circumferences (HC) were measured.

Body shape from childhood to recruitment was assessed in the lifestyle questionnaire using a validated scale of silhouette pictograms^15,16^ used in similar studies^9^, adapted from Sørensen *et al*.^17^. Women were invited to choose, among 6 drawings (ranging from 1 to 6, leanest to largest), the one that best represented their body shape between ages 6 to 11 (childhood), 12 to 18 (adolescence), 19 to 25, before their first pregnancy, from age 26 to one year before inclusion, and at inclusion.

### Statistical analyses

Main characteristics of the population were described using mean and standard deviation (continuous variables) or frequency (categorical variables). Anthropometric measures of interest were height, leg length (height minus sitting height), weight (kg), waist and hip circumferences, BMI (weight (kg)/height (m^2^)), and waist-to-hip ratio. Silhouettes at different ages were also considered. To ensure that the number of women in each category would be appropriate for the statistical analysis, silhouettes were grouped as follows: between ages 6 to 11, 12 to 18, 19 to 25, and before their first pregnancy, categories were 1, 2, 3, and ≥4; between age 26 and one year before inclusion and at inclusion, categories were 1–2, 3, 4, and ≥5.

Trajectories of body shape were estimated based on silhouettes between ages 6 and 11, 12 to 18, and 19 to 25 (before categorization). Silhouettes at older ages were not considered because not all women were parous and because of the large range of ages represented in each category. Using a group-based trajectory modelling approach (SAS Proc TRAJ)^18^, we followed existing recommendations^19^ to select the model providing the best fit to the data. We successively examined models with one, two, three and four trajectories, after selecting trajectories shapes (quadratic or linear) by examining significance of the model coefficient estimates and the Bayesian Information criterion (BIC) of each model. The BICs of the four models obtained were compared using log Bayes factor, considering the minimum number of women assigned to each trajectory, and examining mean posterior group membership probabilities for each trajectory (expected to be greater than 0.80)^19^.

Odds ratios (OR) for risk of breast cancer and their associated 95% confidence intervals (CI) were estimated using conditional logistic regression analyses. Anthropometric measures were examined as continuous variables and in tertiles (T), defined on the distribution of control participants. For tests of linear trend across tertiles, participants were assigned the median value in each tertile and the corresponding variables were modelled as a continuous term. For silhouettes (ordinal variables), linear trends were tested by including the variables as a continuous term in the model. A combination of BMI categories (normal weight, 18.5 to 24.9; overweight 25 to 29.9; obese ≥30^20^) and WC circumference categories (≤/>88 cm^21,22^) was also examined in order to investigate the role of central obesity across different weight categories.

For all analyses, a model including only matching factors (age, district of residence, health institution) was initially used (crude model). To identify potential confounders, models of the variables of interest were adjusted separately for each potential confounder (continuous and tertiles), and estimates obtained were compared with estimates from unadjusted models. Only variables that changed the risk estimates by more than 10% were retained in the multivariate model. Variables that were ultimately included were: education (≤primary/secondary/>secondary), history of benign breast disease (yes/no), family history of breast cancer (yes/no), total of daily hours of physical activity (tertiles), number of full-term pregnancies (0/1/≥2), age at first full-term pregnancy (tertiles/nulliparous), cumulated duration of breastfeeding (</≥12 months), age at menarche </≥ 12 years old), smoking status (current/former/never), type 2 diabetes (yes/no), daily alcohol intake (tertiles/non-consumer). Except for height and leg length, models were additionally adjusted for height (tertiles). Lastly, we examined the association of WC and HC independently from overall obesity using a mutually adjusted model including BMI and residuals of WC and HC on BMI, calculated from two separate linear regression models with BMI as the independent variable and WC or HC as the dependent variable^23^.

Missing values on potential confounders were imputed to the median (age at menarche and at first full-term pregnancy) or mode (number of full-term pregnancies) as they represented less than 5% of the observations on the population^24^.

For cases with currently available information on immunohistochemistry (n = 287), we stratified analyses of anthropometric factors by estrogen receptor status, and triple-negative tumor. Additionally, we investigated whether the associations observed differed by size of the tumor at diagnosis (≤2 cm, >2 cm). All statistical tests were two-sided. Analyses were conducted using SAS software for Windows (version 9.4, Copyright ^©^ 2017, SAS Institute Inc.).

## Results

### Characteristics of the population at inclusion

Compared with controls, a higher percentage of cases had completed secondary school (cases: 37.7%; controls: 24.1%) and never had children (cases: 18.0%; controls: 9.6%). Cases had a first full-term pregnancy at older ages (cases: 24.0 years; controls: 21.7 years) and breastfed for shorter periods their children (breastfeeding for at least 12 months: cases: 39.2%; controls: 60.8%), had more frequently a history of benign breast disease (cases: 36.5%; controls: 12.8%) and smoked less than controls (ever smokers: cases: 47.7%; controls: 54.4%) (Table 1). Controls had a mean height of 1.57 m (1.58 m in cases), a mean weight of 71.4 kg (66.4 kg in cases), a mean WC of 93.1 cm (90.3 cm in cases), a mean HC of 106.3 cm (103.6 cm in cases), and a mean BMI of 28.8 kg/m^2^ (26.5 kg/m^2^ in cases). Average WHR was 0.87 for cases and controls (Table 1). ER+ tumors represented 71.4% of the cases with available immunohistochemistry (n = 287), and 67.6% of these tumors were PR+, while 21.6% of the tumors were TN (Table 1).

**Table 1:** Main characteristics of the population.

Variable	N	Controls	Cases
		N = 406	N = 406
		Mean (SD) or N (%)	Mean (SD) or N (%)
**Age** (**years**)	812	38.6 (5.2)	38.7 (5.1)
**Age at menarche** (**years**)	812	12.6 (1.8)	12.5 (1.7)
**Age at first full-term pregnancy, for parous women** (**years**)	700	21.7 (5.0)	24.0 (5.8)
**Number of children**	812		
0		39 (9.6)	73 (18.0)
1		78 (19.2)	119 (29.3)
≥2		289 (71.2)	214 (52.7)
**Cumulated duration of breastfeeding ≥ 12 months** (**yes**)	812	247 (60.8)	159 (39.2)
**History of benign breast disease** (**yes**)	812	52 (12.8)	148 (36.5)
**Family history of breast cancer in first-degree relatives** (**yes**)	812	20 (4.9)	25 (6.2)
**Daily alcohol intake, among consumers** (**g/day**)	682	2.1 (4.2)	2.3 (4.5)
**Daily vigorous and moderate physical activity** (**hours/day**)	812	4.5 (3.2)	3.4 (3.4)
**Education level**	812		
<primary school		92 (22.7)	55 (13.5)
secondary school		216 (53.2)	198 (48.8)
>secondary school		98 (24.1)	153 (37.7)
**Ever smoker** (**yes**)	812	221 (54.4)	194 (47.7)
**Diagnosis of type 2 diabetes** (**yes**)	812	25 (6.2)	13 (3.2)
**Height** (**m**)	809	1.57 (0.06)	1.58 (0.06)
**Leg length** (**cm**)	806	73.4 (5.6)	73.9 (5.6)
**Weight** (**kg**)	811	71.4 (16.2)	66.4 (13.0)
**Waist circumference** (**cm**)	806	93.1 (14.8)	90.3 (12.3)
**Hip circumference** (**cm**)	807	106.3 (13.3)	103.6 (10.0)
**BMI** (**kg/m**^2^)	809	28.8 (6.0)	26.5 (5.0)
**Waist-to-hip ratio**	806	0.87 (0.08)	0.87 (0.08)
**Trajectory**	812		
T1 ‘Increase at puberty'		115 (28.3)	125 (30.8)
T2 ‘Constant increase - lean'		239 (58.9)	234 (57.6)
T3 ‘Constant increase - overweight'		52 (12.8)	47 (11.6)
**Tumor characteristics**	287^a^		
Estrogen receptor positive (irrespective of other receptors)		—	205 (71.4)
Progesterone receptor positive (irrespective of other receptors)		—	194 (67.6)
HER2 positive (irrespective of other receptors)		—	47 (16.4)
Triple negative (TN): ER−/PR−/HER2−		—	62 (21.6)
*Of which Basal-like* (*TN and EGFR+* and*/or CK5/6+*)		—	*55* (*19.2*)

Abbreviations: BMI, body mass index; CK5/6, cytokeratin 5/6; EGFR, epidermal growth factor receptor; ER, estrogen receptor; HER2, human epidermal growth factor receptor 2; PR, progesterone receptor; SD, standard deviation.

^a^Immunohistochemistry is so far available for 287 cases. Percentages given for tumor characteristics are based on these 287 cases.

### Evolution of body shape during the life course

**Figure 1:**
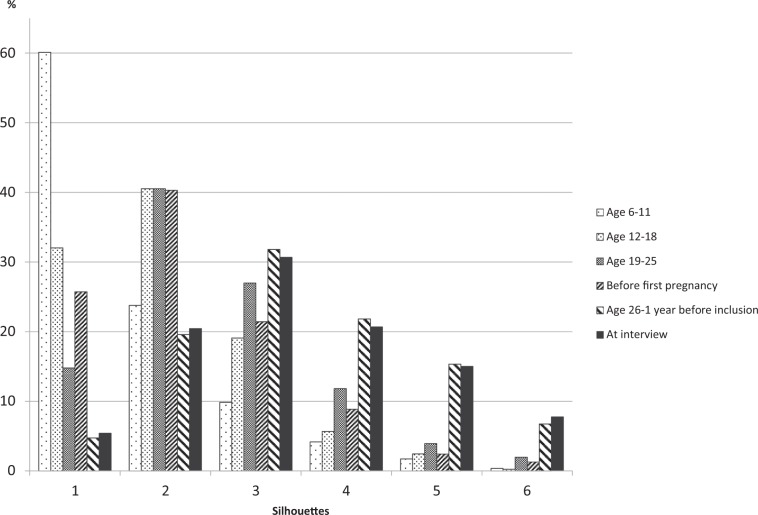
Distribution of body shape by age group in the entire population.

The most frequently chosen silhouettes were silhouette 1 (60.1%) during childhood, silhouette 2 during adolescence (40.5%), from age 19 to 25 (40.5%) and before first pregnancy (40.3%), and silhouette 3 from age 26 to 1 year before interview (31.8%) and at interview (30.7%) (Fig. 1).

**Figure 2:**
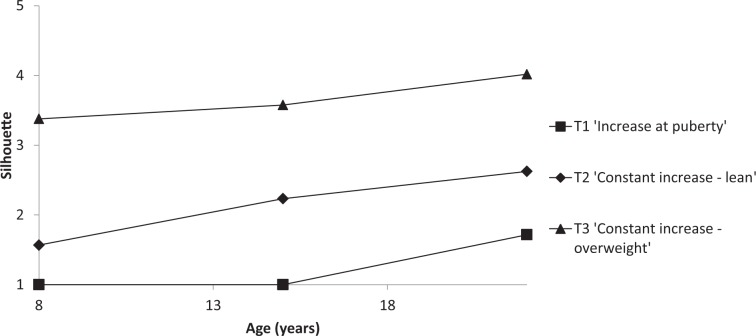
Trajectories of body shape by age.

Three trajectories of body shape evolution were identified (Fig. 2). Two of these trajectories corresponded to a linear model of age: T1, which was named ‘Increase at puberty’, and T3, showing an increase in silhouette starting from an overweight silhouette (named ‘Constant increase – overweight’). The remaining T2 trajectory was based on a quadratic model of age and corresponded to constant increase starting from a lean silhouette (named ‘Constant increase – lean’). Percentages of women in T1, T2 and T3 were 29.6%, 58.3%, and 12.2% respectively, and mean posterior group membership probabilities were 0.92, 0.95, and 0.88, respectively.

### Anthropometry and risk of breast cancer

In the crude model (model 1, Table 2), a positive association was observed between height and risk of breast cancer (OR_T3-T1_ = 1.47, 95% CI = 1.03–2.10, P-trend = 0.03; OR per 10 cm = 1.27, 95% CI = 1.01–1.59), whereas negative associations were observed with weight (OR_T3-T1_ = 0.45, 95% CI = 0.31–0.64, P-trend < 0.01; OR per 10 kg = 0.79, 95% CI = 0.71–0.87), WC (OR_T3-T1_ = 0.59, 95% CI = 0.40–0.87, P-trend = 0.01; OR per 10 cm = 0.83, 95% CI = 0.73–0.93), HC (OR_T3-T1_ = 0.64, 95% CI = 0.45–0.92, P-trend = 0.01; OR per 10 cm = 0.82, 95% CI = 0.72–0.93), and BMI (OR_T3-T1_ = 0.36, 95% CI = 0.25–0.53, P-trend < 0.01; OR per kg/m^2^ = 0.92, 95% CI = 0.90–0.95). After adjustment for potential confounders, all variables but height remained associated with breast cancer risk (BMI: OR_T3-T1_ = 0.36, 95% CI = 0.22–0.59, P-trend < 0.01; OR per 1 kg/m^2^ increment = 0.93, 95% CI = 0.89–0.96; weight: OR_T3-T1_ = 0.33, 95% CI = 0.20–0.54, P-trend < 0.01; OR per 1 kg/m^2^ increment = 0.73, 95% CI = 0.63–0.85; WC (continuous only): OR per 10 cm = 0.81, 95% CI = 0.69–0.96; HC (continuous only): OR per 10 cm = 0.80, 95% CI = 0.67–0.95). No association was observed with leg length (data not shown). In normal weight women (18.5 ≤ BMI < 25 kg/m^2^) women with central obesity (WC > 88 cm) had an increased risk of breast cancer compared to women with normal WC (Model 1: OR = 2.84, 95% CI = 1.44–5.61; Model 2: OR = 3.60, 95% CI = 1.47–8.79). Since almost all obese women had a high WC, it was not possible to investigate the effect of central obesity in this category. Finally, in the mutually adjusted model based on residuals, positive associations were observed with HC (OR = 1.66, 95% CI = 1.13–2.45) and WC (OR = 1.38, 95% CI = 0.98–1.94).

**Table 2:** Odds ratios (OR) and 95% confidence interval (CI) for associations between anthropometric measures at inclusion (continuous and tertiles) and risk of breast cancer, overall.

	Cases/controls	Model 1 - matched^a^	Model 2 - fully adjusted^b^	Model 3 - residuals^c^
		OR (95% CI)	OR (95% CI)	OR (95% CI)
**Height** (**m**)				
Continuous, per 10 cm	403/403	1.27 (1.01–1.59)	1.00 (0.75–1.34)	
<1.54	116/138	1.00 (ref.)	1.00 (ref.)	
1.54–1.59	126/130	1.18 (0.83–1.67)	1.05 (0.67–1.63)	
≥1.60	161/135	1.47 (1.03–2.10)	1.11 (0.71–1.73)	
P-trend		0.03	0.66	
**Weight** (**kg**)				
Continuous, per 10 kg	405/405	0.79 (0.71–0.87)	0.73 (0.63–0.85)	
<63.9	186/135	1.00 (ref.)	1.00 (ref.)	
63.9–75.8	137/132	0.77 (0.55–1.07)	0.59 (0.37–0.94)	
≥75.9	82/138	0.45 (0.31–0.64)	0.33 (0.20–0.54)	
P-trend		<0.01	<0.01	
**Waist circumference** (**cm**)				
Continuous, per 10 cm	400/400	0.83 (0.73–0.93)	0.81 (0.69–0.96)	1.38 (0.98–1.94)
<86	149/132	1.00 (ref.)	1.00 (ref.)	
86–98.9	155/136	0.97 (0.69–1.36)	1.09 (0.69–1.71)	
≥99	96/132	0.59 (0.40–0.87)	0.66 (0.40–1.09)	
P-trend		0.01	0.12	
**Hip circumference** (**cm**)				
Continuous, per 10 cm	401/401	0.82 (0.72–0.93)	0.80 (0.67–0.95)	1.66 (1.13–2.45)
<100	147/132	1.00 (ref.)	1.00 (ref.)	
100–108.7	157/135	1.05 (0.74–1.49)	1.15 (0.73–1.82)	
≥109	97/134	0.64 (0.45–0.92)	0.68 (0.42–1.10)	
P-trend		0.01	0.09	
**BMI** (**kg/m**^2^)				
Continuous, per 1 unit	403/403	0.92 (0.90–0.95)	0.93 (0.89–0.96)	0.92 (0.89–0.96)
<25.6	191/134	1.00 (ref.)	1.00 (ref.)	
25.6–30.1	140/136	0.71 (0.51–0.98)	0.66 (0.43–1.01)	
≥30.2	72/133	0.36 (0.25–0.53)	0.36 (0.22–0.59)	
P-trend		<0.01	<0.01	
**Waist-to-hip ratio**				
Continuous, per 0.1 unit	400/400	0.90 (0.72–1.13)	0.90 (0.68–1.20)	
<0.84	141/133	1.00 (ref.)	1.00 (ref.)	
0.84–0.90	124/133	0.86 (0.60–1.25)	0.88 (0.55–1.41)	
≥0.91	135/134	0.92 (0.61–1.40)	0.99 (0.58–1.70)	
P-trend		0.65	0.91	
**Combined BMI and WC**^e^				
Normal weight/Normal WC	116/90	1.00 (ref.)	1.00 (ref.)	
Normal weight/High WC	45/12	2.84 (1.44–5.61)	3.60 (1.47–8.79)	
Overweight/Normal WC	46/52	0.68 (0.41–1.13)	0.65 (0.34–1.27)	
Overweight/High WC	105/93	0.89 (0.58–1.37)	1.02 (0.59–1.77)	

Abbreviations: BMI, body mass index; CI, confidence interval; OR, odds ratio.

^a^Odds ratios were estimated by logistic regression conditioned on age (±3 years), city district of residence, and health insurance institution.

^b^Additionally adjusted for education (≤primary/secondary/>secondary), history of benign breast disease (yes/no), family history of breast cancer (yes/no), total physical activity (tertiles), number of full-term pregnancies (0/1/≥2), age at first full-term pregnancy (tertiles/nulliparous/missing), cumulated duration of breastfeeding (</≥12 months), age at menarche (</≥12 years old), smoking status (current/former/never), diabetes (yes/no), daily alcohol intake (tertiles/non-consumer). Except for height and leg length, models were additionally adjusted for height (tertiles), thus only women with available information on height were included in the analyses for model 2.

^c^In model 3, BMI, residuals of waist circumference regressed on BMI and residuals of hip circumference regressed on BMI were included simultaneously, in addition to adjustment variables of model 2.

^d^For test of linear trend across tertiles, participants were assigned the median value in each tertile and the corresponding variable was modelled as a continuous term.

^e^Only women with a BMI ≥ 18.5 kg/m^2^ were included in this analysis. BMI was categorized as normal weight (18.5–24.9), overweight (25–29.9) and obese (≥30) and WC was categorized as normal (≤88 cm) and high (>88 cm).

### Evolution of body shape and risk of breast cancer

No association was observed for body shape during childhood, adolescence or early adulthood and risk of breast cancer, either in the model conditioned on matching factors only nor in the adjusted model (Table 3). Regarding the evolution of body shape between these ages, compared with the T1 trajectory ‘Increase at puberty’, no association was observed in T2 ‘Constant increase – lean’ or T3 ‘Constant increase – overweight’. Negative associations were observed for silhouettes between 26 years old and one year before interview (model 1: OR ≥ 5 vs 1–2 = 0.39, 95% CI = 0.25–0.61, P-trend < 0.01; model 2: OR ≥ 5 vs 1–2 = 0.45, 95% CI = 0.25–0.79, P-trend < 0.01) and at interview (model 1: OR ≥ 5 vs 1–2 = 0.31, 95% CI = 0.20–0.49, P-trend < 0.01; model 2: OR ≥ 5 vs 1–2 = 0.33, 95% CI = 0.19–0.59, P-trend < 0.01).

**Table 3:** Odds ratios (OR) and 95% confidence interval (CI) for associations between body shape from childhood to inclusion and risk of breast cancer, overall.

	Cases/controls	Model 1 - matched^a^	Model 2 - fully adjusted^b^
		OR (95% CI)	OR (95% CI)
**Silhouette between 6 and 11**			
1	256/232	1.00 (ref.)	1.00 (ref.)
2	84/109	0.69 (0.49–0.98)	0.61 (0.39–0.95)
3	40/40	0.92 (0.57–1.48)	0.98 (0.52–1.86)
≥4	26/25	0.93 (0.52–1.65)	1.02 (0.48–2.18)
P-trend		0.39	0.61
**Silhouette between 12 and 18**			
1	133/127	1.00 (ref.)	1.00 (ref.)
2	165/164	0.96 (0.68–1.35)	1.14 (0.73–1.78)
3	77/78	0.95 (0.63–1.43)	0.83 (0.49–1.42)
≥4	31/37	0.80 (0.47–1.37)	1.03 (0.52–2.03)
P-trend		0.47	0.68
**Silhouette between 19 and 25**			
1	66/54	1.00 (ref.)	1.00 (ref.)
2	178/151	0.98 (0.64–1.49)	1.19 (0.70–2.04)
3	92/127	0.59 (0.38–0.93)	0.70 (0.39–1.26)
≥4	70/74	0.79 (0.49–1.27)	1.09 (0.58–2.05)
P-trend		0.05	0.56
**Silhouette before first pregnancy** (**parous women**)			
1	79/77	1.00 (ref.)	1.00 (ref.)
2	128/117	1.05 (0.70–1.56)	0.75 (0.45–1.25)
3	64/68	0.92 (0.57–1.47)	0.74 (0.40–1.36)
≥4	33/42	0.78 (0.45–1.35)	0.57 (0.27–1.19)
P-trend		0.31	0.15
**Silhouette between age 26 and 1 year before inclusion**			
1–2	118/77	1.00 (ref.)	1.00 (ref.)
3	140/114	0.77 (0.52–1.14)	0.97 (0.58–1.60)
4	73/102	0.44 (0.28–0.68)	0.42 (0.24–0.76)
≥5	69/107	0.39 (0.25–0.61)	0.45 (0.25–0.79)
P-trend		<0.01	<0.01
**Silhouette at interview**			
1–2	128/82	1.00 (ref.)	1.00 (ref.)
3	141/108	0.79 (0.53–1.17)	0.82 (0.49–1.35)
4	72/96	0.43 (0.27–0.67)	0.44 (0.25–0.77)
≥5	65/120	0.31 (0.20–0.49)	0.33 (0.19–0.59)
P-trend		<0.01	<0.01
**Trajectory**			
T1 ‘Increase at puberty'	125/115	1.00 (ref.)	1.00 (ref.)
T2 ‘Constant increase - lean'	234/239	0.90 (0.65–1.23)	1.03 (0.68–1.56)
T3 ‘Constant increase - overweight'	47/52	0.82 (0.51–1.33)	0.91 (0.49–1.70)

Abbreviations: CI, confidence interval; OR, odds ratio.

^a^Odds ratios were estimated by logistic regression conditioned on age (±3 years), city district of residence, and health insurance institution.

^b^Additionally adjusted for education (≤primary/secondary/>secondary), history of benign breast disease (yes/no), family history of breast cancer (yes/no), total physical activity (tertiles), number of full-term pregnancies (0/1/≥2), age at first full-term pregnancy (tertiles/nulliparous/missing), cumulated duration of breastfeeding (</≥ 12 months), age at menarche (</≥ 12 years old), smoking status (current/former/never), diabetes (yes/no), daily alcohol intake (tertiles/non-consumer), height (tertiles). Only women with available information on height were included in the analyses for model 2.

### Stratified analyses

Associations of anthropometric factors and risk of breast cancer did not differ by breast cancer subtype (Table 4), although after adjustment for covariates, weight and BMI were negatively associated with ER− tumors only (BMI: ER+: OR per 1 kg/m^2^ = 0.95, 95% CI = 0.90–1.01; ER−: OR per 1 kg/m^2^ = 0.90, 95% CI = 0.82–0.99; TN: OR per 1 kg/m^2^ = 0.92, 95% CI = 0.79–1.06/weight: ER+: OR per 10 kg = 0.83, 95% CI = 0.66–1.03; ER−: OR per 10 kg = 0.60, 95% CI = 0.39–0.92; TN: OR per 10 kg = 0.63, 95% CI = 0.32–1.26). However, evidence for heterogeneity was weak (P-homogeneity ER+ vs ER− = 0.19 for weight, 0.34 for BMI; P-homogeneity TN vs non-TN = 0.22 for weight, 0.88 for BMI; not tabulated).

**Table 4:** Associations between anthropometric measures and risk of breast cancer stratified by tumour subtype.

	Estrogen receptor positive		Estrogen receptor negative		Triple negative	
	Cases/controls	OR^a^ (95% CI)	Cases/controls	OR^a^ (95% CI)	Cases/controls	OR^a^ (95% CI)
Height, per 10 cm	203/203	1.06 (0.68–1.66)	81/81	1.03 (0.45–2.39)	61/61	1.09 (0.32–3.78)
Leg length, per 10 cm	202/202	0.93 (0.50–1.72)	79/79	1.01 (0.33–3.10)	60/60	1.02 (0.26–3.96)
Weight, per 10 kg	203/203	0.83 (0.66–1.03)	81/81	0.60 (0.39–0.92)	61/61	0.63 (0.32–1.26)
Waist circumference, per 10 cm	202/202	0.97 (0.75–1.26)	80/80	0.73 (0.47–1.13)	60/60	1.18 (0.58–2.39)
Hip circumference, per 10 cm	202/202	0.86 (0.65–1.12)	80/80	0.73 (0.44–1.19)	60/60	2.27 (0.76–6.81)
BMI, per 1 kg/m^2^	203/203	0.95 (0.90–1.01)	81/81	0.90 (0.82–0.99)	61/61	0.92 (0.79–1.06)
Waist-to-hip ratio, per 0.1 unit	202/202	1.37 (0.85–2.20)	80/80	0.68 (0.27–1.69)	60/60	0.63 (0.19–2.09)

Abbreviations: BMI, body mass index; CI, confidence interval; OR, odds ratio.

^a^Odds ratios were estimated by logistic regression conditioned on age (±3 years), city district of residence, and health insurance institution and adjusted for education (≤primary/secondary/> secondary), history of benign breast disease (yes/no), family history of breast cancer (yes/no), total physical activity (tertiles), number of full-term pregnancies (0/1/≥2), age at first full-term pregnancy (tertiles/nulliparous/missing), cumulated duration of breastfeeding (</≥12 months), age at menarche (</≥12 years old), smoking status (current/former/never), diabetes (yes/no), daily alcohol intake (tertiles/non-consumer). Except for height and leg length, all models were additionally adjusted for height (tertiles).

No heterogeneity was observed for any of the anthropometric factors when stratifying the analyses by tumor size (≤2 cm, >2 cm) (P-homogeneity ≥0.09 for model 2, results not shown).

## Discussion

In this population of premenopausal women from 4 Latin American countries, weight, waist and hip circumference, and BMI were negatively associated with risk of breast cancer, as was body shape from age 26 to study enrollment. However, in normal weight women, women with central obesity had an increased risk of breast cancer compared to women with normal WC, and, in all women, when adjusting for BMI, HC and WC were positively associated with risk of breast cancer. Height, leg length and WHR at recruitment were not associated with risk of breast cancer, neither was body shape at young ages and trajectories of body shape evolution. Stratified analyses showed no evidence of heterogeneity by subtypes of breast cancer. These findings point towards a role for body size in premenopausal breast cancer development in Latin American women, as reported in different populations.

### Overall adiposity

The only study on anthropometry and premenopausal breast cancer in Latin America (the CAMA case-control study, Mexico, 415 cases) showed that obese (BMI ≥ 30 kg/m^2^) women had a decreased risk of breast cancer compared with those having a BMI lower than 25 kg/m^2^ ^9^, consistent with our findings. Among Hispanic premenopausal women in the US, two studies reported an negative association between BMI and breast cancer risk, only in ER+PR+ tumors^25^ or in ER− tumors^26^, while a third one showed no association^27^. Our findings are in line with the negative association between adiposity and premenopausal breast cancer that has been reported in other populations^4^, as well as for genetically predicted BMI^28^, but are not in favor of an association restricted to hormone-receptor positive tumors^8^, although sample size in subgroups is limited in this analysis. This negative association between overall adiposity and premenopausal breast cancer is not well understood, but some mechanisms have been suggested, such as a reduced exposure to estrogens resulting from anovulation and abnormal hormone profiles induced by excess adiposity^29,30^.

### Central adiposity

Evidence from the CAMA study suggested a negative association between WC, HC and WHR and risk of premenopausal breast cancer^9^. We replicated these negative associations with HC and WC, but not WHR. Interestingly, when models were adjusted for current BMI, in the CAMA study, the negative association with WC (raw measure, not residuals) persisted^9^. In contrast, our results showed a positive association with HC (residuals) and with WC (residuals) when adjusted for BMI. The interpretation of WC and residuals of WC is slightly different, since WC is a measure of central adiposity that correlates with overall adiposity as measured by BMI (which might induce collinearity issues when included in the same model), while residuals of WC should be interpreted as a measure of central adiposity independent of overall adiposity^23^. Residuals of HC, when adjusted for BMI, are an indicator of the gluteofemoral lean mass, fat accumulation, and bone structure of the pelvis^23^. These findings are consistent with the increased risk in normal-weight women with central obesity observed in this study. Therefore, our data suggest that, although overall adiposity is negatively associated with risk of breast cancer, central adiposity and gluteofemoral adiposity show a specific positive association with breast cancer. These results are in line with the positive association with WC and risk of premenopausal breast cancer (independent of BMI) reported among Hispanic women living in the US in the Breast Cancer Health Disparities Studies^25^. A positive association between WC and risk of cancer has already been observed in other populations when adjusting for BMI^4^ and in normal-weight women^31^, and high HC has also been associated with increased risk of breast cancer^32^, in particular ER-PR- tumors^33^. The interpretation of such opposite associations between different measures of adiposity remains complex. Strong correlations between WC and high insulin levels^34^ could explain the link between higher WC and increased breast cancer risk^35^, while the specificities of the gluteofemoral adipose tissue, for which HC is a marker^36^, such as secretion of leptin^37^, might be involved in the association with HC. However, a better understanding of the parameters measured by HC is needed for further interpretation of these findings. In particular, HC and WC are only proxies for body fat distribution, and techniques such as DEXA or impedance, which provide information on body composition and fat distribution, would be helpful in disentangling these associations.

### Height

In contrast with observations in Caucasian women and in the CAMA study, we did not observe any association between height and breast cancer risk^5,9^. In our study, neither height nor leg length, a marker of pubertal growth^38^, were associated with risk of breast cancer. It is possible that the absence of association we observed resulted from a reduced variability in our sample population compared with women from Caucasian populations^39,40^.

### Evolution of body shape

The negative associations reported for larger body shape during adult life and at interview are in line with our findings on overall adiposity measured by BMI, and with results in Latin America^9^ or Hispanic women^41^. Although a negative association between body size in childhood/adolescence and risk of breast cancer in young women has been frequently reported in Caucasian populations^42^, in our study as in the premenopausal women of the CAMA study^9^, body shape early in life was not associated with breast cancer risk. Interestingly, a study on early-life body shape in Hispanic women in the US^43^ showed that, while changes in silhouettes was not associated with risk of premenopausal breast cancer risk, an negative association was observed with changes in weight at ages 10, 15 and 20 (heavier/same/lighter than peers) and that this association tended to be stronger in US-born than in foreign-born women. Thus, the absence of associations in Latin American women could indicate that the influence of early-life body size on breast cancer risk varies between populations, or could also suggest that the silhouette scales, although validated in European^15^ and North American^16^ populations, might not be a tool fully adapted to other populations.

The three trajectories that we obtained between childhood and early adulthood are comparable with some of the five trajectories obtained from the data of the CAMA study (groups 2, 3 and 4). However, both pre and postmenopausal women were included when modelling the trajectories. Similarly to our work, no association was observed with any of the trajectories in premenopausal women, and, although in pre and postmenopausal women combined, positive associations were observed when considering a very lean and stable body shape at the three ages as a reference, the risk estimate were very close for these three groups^9^. These associations are likely to be driven by the positive associations with postmenopausal breast cancer. Although weight gain has been associated with postmenopausal breast cancer risk, the evidence is limited for premenopausal women^4^ and studies in Hispanics in the US have yielded inconsistent results^44^. In addition, our trajectories focused on change in body shape during childhood and adolescence, not adulthood, which makes comparison difficult with most existing studies.

Our study is the largest ongoing multicentric population-based case-control study of premenopausal breast cancer in Latin America. Strengths of this work include the use of standardized anthropometric factors measured by trained clinicians on incident cases, the use of standardized tools and questionnaires across centers to collect information on various current and past lifestyle factors, and the centralization of immunohistochemistry analyses, thus limiting the inter-laboratory variability. Still, some limitations must be acknowledged, such as the subjective nature of body shape evaluation based on silhouettes, although this tool has been validated and has proven to be reliable for lifelong approaches of body size^16^. Also, anthropometry does not provide in-depth information on fat distribution, as would DEXA, or on body composition, which is why bioelectrical impedance measures are being implemented in the recruitment process of PRECAMA in Brazil. In addition, because of the case-control design of the study, some of the associations observed could result from reverse causation. Our design might also have impacted the trajectories obtained in this work, which is why the trajectories described are specific to this study and should not be generalized. However, the recruitment of cases before the start of any treatment which may result in weight loss, as well as the consistency of the negative association observed when considering measures of current body size, silhouette at interview and silhouette up to one year before interview does not support this hypothesis. Also, the negative association observed with BMI did not vary by size of the tumor. Finally, the relatively small sample size because of the ongoing recruitment resulted in limited power in some subgroups in the stratified analyses, even if the study is the largest ongoing effort in this population.

In summary, our findings suggest that the negative association between adiposity and risk of breast cancer before menopause established in other populations is also observed in women in Latin America, regardless of breast cancer subtype. More research is needed to better understand the underlying mechanisms of this relationship, and to characterize the distribution of body fat in this understudied population of premenopausal Latin American women.

## Data Availability

PRECAMA data and biospecimens are available for investigators who seek to answer important questions on health and disease in the context of research projects that are consistent with the legal and ethical standard practices of IARC/World Health Organization (WHO) and the PRECAMA Centres. The primary responsibility for accessing the data belongs to the PRECAMA centres that provided them. The use of a random sample of anonymised data from the PRECAMA study can be requested by contacting the corresponding author. The request will then be passed to members of the PRECAMA Steering Committee for deliberation.
